# Changes in Equity in Out-of-pocket Payments during the Period of Health Care Reforms: Evidence from Hungary

**DOI:** 10.1186/1475-9276-11-36

**Published:** 2012-07-24

**Authors:** Petra Baji, Milena Pavlova, László Gulácsi, Wim Groot

**Affiliations:** 1 Health Economics and Health Technology Assessment Research Centre, Corvinus University of Budapest, Budapest, Hungary; 2 Center for Public Affairs Studies Foundation, Budapest, Hungary; 3 Department of Health Services Research, CAPHRI, Maastricht University Medical Center; Faculty of Health, Medicine and Life Sciences, Maastricht University, Maastricht, The Netherlands; 4 Topinstitute Evidence-Based Education Research (TIER), Maastricht University, Maastricht, The Netherlands

**Keywords:** Household expenditure, Health care reforms, Equity, Kakwani index, Hungary

## Abstract

**Background:**

At the beginning of 2007, health care reforms were implemented in Hungary in order to decrease public expenditure on health care. Reforms involved the increase of co-payments for pharmaceuticals and the introduction of co-payments for health care services.

**Objective:**

The objective of this paper is to examine the progressivity of household expenditure on health care during the reform period, separately for expenditures on pharmaceuticals and medical devices, as well as for formal and informal patient payments for health care services.

**Methods:**

We use data on household expenditure from the Household Budget Survey carried out by the Central Statistical Office of Hungary. We present household expenditure as a percentage of household income across different income quintiles and calculate Kakwani indexes as a measure of progressivity for a four years period (2005–2008): before, during and after the implementation of the health care reforms.

**Results:**

We find that out-of-pocket payments on health care are highly regressive in Hungary with a Kakwani index of −0.22. In particular, households from the lowest income quintile spend an about three times larger share of their income on out-of-pocket payments (6–7 %) compared to households in the highest income quintile (2 %). Expenditures on pharmaceuticals and medical devices are the most regressive types of expenditure (Kakwani index −0.23/-0.24), and at the same time they represent a major part of the total household expenditure on health care (78–85 %). Informal payments are also regressive while expenditures on formal payments for services are the most proportional to income. We find that expenditures on formal payments became regressive after the introduction of user fees (Kakwani index −0.1). At the same time, we observe that expenditures on informal payments became less regressive during the reform period (Kakwani index increases from −0.20/-0.18 to −0.12.)

**Conclusions:**

More attention should be paid on the protection of low-income social groups when increasing or introducing co-payments especially for pharmaceuticals but also for services. Also, it is important to eliminate the practice of informal payments in order to improve equity in health care financing.

## Introduction

Out-of-pocket payments already represent a non-negligible part of total health care expenditure in Central and Eastern European countries [1]. However, reform arrangements intended to decrease public expenditure on health care often lead to the further increase of out-of-pocket payments in health care financing. According to the results of previous studies, out-of-pocket payments are a regressive means of financing health care (e.g. [2-4]). Thus, households with lower income spend a relatively higher share of their income on health care than those with higher income. This is in conflict with the European health policy targets, which argue that the ability to pay rather than the health status should determine health care payments. In other words, the distribution of health care payments should be in line with the distribution of households’ income rather than with the actual consumption of health care (e.g. [1,3]). Reform arrangements, which lead to the increasing role of out-of pocket payments in health care financing, might lead to even higher differences between income groups, inducing a greater burden falling on the worse-off households [5-7].

In this paper we analyze the progressivity of households’ out-of-pocket payments for health care in Hungary, where at the beginning of 2007, health care reforms were carried out in order to decrease public expenditures on health care. Besides structural and regulative arrangements, the austerity measures resulted in increased patient co-payments for pharmaceuticals and in the introduction of co-payments for using health care services.

The aim of our paper is to examine the progressivity of household expenditure on health care during a four years period (2005–2008): before, during and after the implementation of the health care reforms. We are particularly interested in whether we can observe significant changes in the distribution of household expenditure on health care across income groups after the implementation of the reforms. For the analysis, we use data from the Household Budget Survey carried out by the Central Statistical Office of Hungary ^a^. We consider total household expenditure on health care as well as its comprising elements, namely household expenditures on pharmaceuticals and medical devices, and on formal and informal patient payments for health care services. Kakwani indexes by types of expenditures are estimated to indicate the progressivity of household expenditure on health care (see [8,9]). Our study provides evidence on how austerity measures in the health care system, such as those carried out in Hungary at the beginning of 2007, can affect the progressivity of households’ out-of-pocket payments and whether the changes can lead to a relatively greater burden on lower income households. Our results are relevant to countries with a similar structure of health care financing, facing similar challenges, especially to countries in the Central and Eastern European region.

## Background: Overview of the reforms in the Hungarian health care sector in 2006–2007

In Hungary the mandatory health insurance system is funded by income-related social health insurance contributions. The contributions of those who are eligible for services, but are not obligated to pay contributions (e.g. children under the age 18, students, pensioners, disabled, women on maternity leave) are covered by the state budget.

The National Health Insurance Fund Administration (NHIFA) is the single health insurance payer, which administers the Health Insurance Fund (HIF). HIF covers the recurrent costs of health services, and finances certain cash benefits, such as sick pay. Capital costs are covered by the local and the central governments. The HIF is divided into more than 30 sub-budgets according to the type of service (e.g. acute inpatient care, chronic inpatient care and outpatient specialist care). Primary care is mostly organized based on private practices having a gate-keeping role. Thus, a referral is needed to visit a specialist except for some services (e.g. dermatological, gynaecological, laryngological, ophthalmological psychiatric services). The vast majority of polyclinics and hospitals are owned by municipalities or the state. Primary care is reimbursed by the NHIFA on a capitation base, while a fee-for-service point system works as a basis for financing out-patient specialist care. Inpatient-care is financed based on diagnosis-related groups [10-12].

The total health expenditure in Hungary accounts for 7.4 % of the GDP in 2009, lower than the OECD average (9.7 %). The total health expenditure per capita is 1,511 USD, which is less than half of the OECD average (3361 USD). Public expenditure accounts for 69.7 % of total health spending [13].

The NHIFA has been facing continuous deficits since its foundation in 1993. The deficit varied from 3.4 % of the total revenue of the Fund in 1994, to 31.2 % of the total revenue in 2005. The cumulative deficit amounted to 1,500 billion HUF (6 billion EUR) in 2005, which is equal to the annual budget of the Fund [14]. The deficit of the NHIFA has led to an increased burden on the state budget as the government is obliged to cover any shortfall in the Health Insurance Found, and shortfalls in the HIF appear in the government budget deficit [10].

Due to the continuous deficit of the NHIFA, the health care system was one of the fields where the Hungarian government had to consider reforms as a part of the Convergence Program of Hungary at the end of 2006 [15]. The objective of the Convergence Program was to contain the government deficit and meet the Maastricht criteria of the EU for joining the Euro zone (i.e. the ratio of the annual government deficit to gross domestic product must not exceed 3 % at the end of the preceding fiscal year and the total state debt must not go beyond 60 % of the GDP [16]). The resulting austerity measures in the health care system in 2006–2007 aimed to decrease public health care expenditure as a percentage of GDP. The reforms affected the regulation, structure and financing of the health care system. Reforms involved the increase of patient co-payments for pharmaceuticals as well as the introduction of co-payments for the use of public health care services, and co-insurance in the case of a free choice of physician which led to the increase of the role of out-of-pocket patient payments in health care financing (see Table 1). However, co-payments for health care services were abolished at the beginning of 2008, as a result of a population referendum [17].

**Table 1:** Overview of the Hungarian health care reforms in 2007

***The expansion of contributing payers***	
The reforms aimed to settle the eligibility criteria for the health for insurance coverage. According to the new regulations, the contributions of those who are eligible for services, but were not obligated to pay contributions (children under the age 18, students, pensioners, disability-pensioners, women on maternity leave) is to be covered by the state budget. Those who do not belong to these categories and who were either employees or self-employed workers are obliged to pay insurance contributions. The payment obligations have also been expanded to dependent family members and agricultural workers. According to the NHIFA’s estimations, the status of more than 1 million citizens in the NHIFA register was uncertain before the reform [18].	
***Changes in health insurance coverage***	
*The introduction of co-payments.* Co-payments for public health care services (called “visit fees”) were introduced in February, 2007 (~ 1.1 euro per visit and per day hospitalization). Children and some patients with chronic conditions were exempted. The revenues from these co-payments were retained by the health care provider. The aim was to make consumers more cost-conscious and to regulate demand for public health care services, as well as to eradicate informal patient payments in Hungary [17,46].	
*Restricted choice of patients to use health care services.* The government also aimed to enforce a system of referrals in the public health care sector. According to the regulation out-patient and in-patient care could only be accessed based on a referral issued at the lower levels of the health care system. In addition, patients’ choice of health care provider has been restricted. Patients could be admitted only to 2-3 hospitals in the region where the patients are living. Higher co-payments (30 percent of the hospital cost) should be paid, if patients wanted to attend hospitals (or physicians) in another region. (see Act 1997/ LXXXIII.)	
***A new act on pharmaceuticals***	
The Act on the Secure and Efficient Supply of Pharmaceuticals and Medical Aids and on the General Rules of Pharmaceutical Trade was adopted by Parliament in November 2006 (Act2006./XCVIII.). This reform was expected to reduce public expenditure on pharmaceuticals by 1) enforcing the role of patients in financing (i.e. by decreasing subsidies on pharmaceuticals) as well as the role of pharmaceutical companies (by the introduction of risk sharing mechanisms between payer and the pharmaceutical companies and the increasing taxes on promotion), 2) supporting generics by regulating the drug prescription system; 3) and to create price-competition between pharmaceutical companies by the liberalisation of the pharmaceutical market.	
*Decreasing subsidies and increasing co-payments.* NHIFA reimburses drugs in three categories: 1) Fully reimbursed drugs 2) Indication dependent drugs 3) Normative reimbursement. The measure of the subsidies is a defined percentage of the negotiated gross price of the medicine. In 2007 reimbursements on pharmaceuticals were decreased. First, for drugs belonging to the category of normative reimbursement, the reimbursement rates have been decreased from 50 % to 25 %; from 70 % to 55 %; and from 90 % to 85 %. For drugs in the category of indication dependent pharmaceuticals the reimbursement rate of 90 % was reconsidered, and replaced by three subsidy categories (50, 70, 90 %). In the third category, where drugs are 100 % subsidized, a minimum 300 HUF co-payment per box was introduced [20].	
***Structural reforms***	
The new system of high priority and territorial hospitals was established in April, 2007 based on the Act . 2006./CXXXII. In total, 77 territorial hospitals and 37 high priority hospitals were set up. Structural reforms concerned the decease of the number of hospital beds in inpatient care as well. Acute bed capacity was cut by 16 000 beds (~ 27 %), while chronic bed capacity increased by 7500 (~31 %) in 2007 [21].	
***The establishment of Health Insurance Supervisory Authority***	
The Health Insurance Supervisory Authority (HISA) was established in December, 2006 to monitor contracts between the NHIFA and the providers. The Authority was also responsible for investigating patients’ complaints.	

*Note:* Beyond these changes, the transformation of the health insurance system was planned as well. However, the idea of replacing the single-payer insurance model by competing Health Insurance Management Funds never materialized in practice. The Act on Health Insurance Management Funds was revoked by Parliament in May 2008.

As a result of the reform arrangements, total health care expenditure as a percentage of GDP decreased from 8.1 % in 2006 to 7.4 % in 2007. Public health care expenditure decreased compared to the year before by 1.8 % in nominal terms and 9.1 % in real terms. In fact, the share of public health care expenditure dropped from 72.5 % to 70.3 % [13]. Also, the NHIFA registered savings in 2007. During this period, the NHIFA’s expenditure on pharmaceuticals decreased by 17 % due to the cost-containment measures such as the increase of patient co-payments for pharmaceuticals [20,22,23].

## Methods: data and analysis

We use data on household expenditure from the Household Budget Survey carried out by the Central Statistical Office of Hungary for the years 2005, 2006, 2007 and 2008. The Central Household Budget Survey (HBS) provides detailed information on yearly expenditure on housing conditions and consumer durables according to the detailed grouping of Classification of Individual Consumption by Purpose (COICOP) international nomenclature ^b^. It also provides data on the social-demographic characteristics of the household.

The data collection consists of two parts: 1/12 of the selected households (about 750 households) keep a diary on their expenses and income for a month followed by a retrospective interview about their income and exceptional expenditures at the next year after the reference year. ^c^ The dataset contains data for 9058 households in 2005, 8975 households in 2006, 8547 households in 2007, and 7650 households in 2008.

In this analysis, we examine four types of household expenditure on health care:

expenditures on pharmaceutical products, medical aids and other medical products (appliances and equipments) – henceforth called as pharmaceuticals and medical devices.

formal payments for public and private health care services (incl. fees for GPs, outpatient physicians, dental care and hospital services both in the private and the public sector, services of laboratories and x-ray centers, ambulance services, services of freelance nurses, midwives and acupuncturists, chiropractors, optometrists and various types of therapists)

informal payments for health care services (informal payments for GP, out-patient and in-patient care, dentist and ambulance);

total health care expenditure (incl. the sum of the first three types of expenditure described above).

We present these types of expenditures by income quintiles as a percentage of the net yearly household income (also available from the HBS survey). To examine the progressivity of household expenditure on health care, we also calculate the Kakwani index. Kakwani indexes are estimated using the regression formula suggested by Kakwani et al. (1997) [9].

This index is widely used for measuring progression in taxation and also, the progression of out-of-pocket payments [2-4]. Recent applications of the index in health care can be found for example in Hanley et al. (2008) and Smith (2010) [24-26]. The index is defined, as twice the area between the payments’ concentration curve and the income (Lorenz) curve. The value of Kakwani index ranges from −2 to 1. A negative value indicates regressive payments, i.e. in households with lower income the share of expenditure as a percentage of income is higher than in households with higher income. A positive value indicates a progressive distribution, i.e. in households with lower income the share of expenditure is lower than in households with higher income. In the case of proportionality, the concentration curve coincides with the Lorenz curve and the Kakwani index is zero [27].

## Results

Table 2 shows the descriptive statistics on the different types of health care expenditure as well as the net yearly household income of the households by income quintiles during the period 2005–2008. In that period, total household expenditure on health care increased from 3.7 % to 4.4 % of the net household income. This share varies between 2.1-2.5 % of the household income in the highest income quintile to 6.1-7.3 % of the household income in the lower income quintiles.

**Table 2:** Household expenditure on health care

**Year**	**Quintile**	**2005**		**2006**		**2007**		**2008**	
**Expenditure**		**mean**	**sd**	**mean**	**sd**	**mean**	**sd**	**mean**	**sd**
**Income (Thousand HUF)**	1^st^	920	213	951***	245	889***	**208**	882	202
	2^nd^	1 539	159	1 586***	159	1 468***	**160**	1 442***	165
	3^rd^	2101	175	2157***	174	2 020***	**166**	1 972***	161
	4^th^	2 790	239	2 840***	238	2 686***	**227**	2 609***	214
	5^th^	4 622	1 802	4 627***	1 992	4 333***	**1 726**	4 062***	1 171
	Total	2 394	1 518	2 433***	1 556	2 279***	**1 426**	2 193***	1 225
**Pharmaceuticals and medical devices (% of income)**	1^st^	4.96 %	5.41 %	5.29 %	6.75 %	6.00 %***	**6.96 %**	6.56 %**	8.05 %
	2^nd^	3.53 %	4.20 %	3.43 %	4.10 %	4.10 %***	**4.67 %**	4.40 %*	5.02 %
	3^rd^	2.38 %	3.02 %	2.42 %	2.95 %	3.03 %***	**3.87 %**	3.26 %*	3.73 %
	4^th^	1.90 %	2.34 %	1.89 %	2.15 %	2.26 %***	**2.69 %**	2.33 %	2.75 %
	5^th^	1.54 %	1.72 %	1.47 %	1.67 %	1.75 %***	**2.14 %**	1.97 %***	2.20 %
	Total	2.86 %	3.80 %	2.90 %	4.19 %	3.43 %***	**4.66 %**	3.71 %***	5.10 %
**Formal payments (% of income)**	1^st^	0.62 %	2.36 %	0.77 %	6.04 %	**0.91 %**	**2.86 %**	0.49 %***	2.06 %
	2^nd^	0.42 %	1.36 %	0.45 %	1.56 %	0.67 %***	**1.57 %%**	0.43 %***	1.49 %
	3^rd^	0.50 %	1.47 %	0.36 %***	1.10 %	0.61 %***	**1.57 %**	0.46 %**	2.12 %
	4^th^	0.41 %	1.15 %	0.45 %	1.49 %	**0.52 %**	**1.64 %**	0.49 %	1.70 %
	5^th^	0.48 %	1.28 %	0.41 %*	0.95 %	0.49 %**	**1.26 %**	0.42 %	1.27 %
	Total	0.49 %	1.58 %	0.49 %	2.95 %	0.64 %***	**1.87 %**	0.46 %***	1.76 %
**Informal payments (% of income)**	1^st^	0.51 %	1.50 %	0.56 %	1.92 %	0.26 %***	**0.81 %**	0.28 %	1.37 %
	2^nd^	0.43 %	1.59 %	0.38 %	1.13 %	0.23 %***	**0.70 %**	0.29 %**	1.01 %
	3^rd^	0.27 %	0.71 %	0.29 %	1.38 %	0.19 %***	**0.67 %**	0.18 %	0.61 %
	4^th^	0.25 %	0.78 %	0.22 %	0.66 %	0.15 %***	**0.52 %**	0.19 %	1.65 %
	5^th^	0.18 %	0.61 %	0.20 %	0.62 %	0.14 %**	**0.63 %**	0.10 %**	0.36 %
	Total	0.33 %	1.13 %	0.33 %	1.25 %	0.19 %***	**0.67 %**	0.21 %	1.11 %
**Total (% of income)**	1^st^	6.10 %	6.60 %	6.62 %*	11.54 %	**7.18 %**	**8.20 %**	7.34 %	9.13 %
	2^nd^	4.38 %	5.10 %	4.26 %	4.89 %	5.00 %***	**5.32 %**	5.11 %	5.87 %
	3^rd^	3.14 %	3.72 %	3.07 %	3.69 %	3.82 %***	**4.54 %**	3.90 %	4.53 %
	4^th^	2.56 %	2.96 %	2.56 %	2.95 %	2.93 %***	**3.42 %**	3.01 %	3.86 %
	5^th^	2.20 %	2.46 %	2.08 %	2.21 %	2.38 %***	**2.81 %**	2.50 %	2.75 %
	Total	3.68 %	4.65 %	3.72 %	6.28 %	4.26 %***	**5.48 %**	4.37 %	5.93 %

Note: 1 EUR  250 HUF during the examined period; income is indicated on 2007 price level indexed by CPI 2005–2006:+3.9 %; 2006–2007:+0.8 %; 2007–2008:+0.61 %; t-test is used to compare the values between the years (H0:the share equal to the share in the previous year): *p < 0.1; **p < 0.05; ***p < 0.001.

The reform period (year 2007) is indicated by bold letters.

The major cost driver of households’ health care expenditure are the expenditures on pharmaceuticals and medical devices, which represent 78 % of total expenditure in 2005, and this share increases to 85 % in 2008. This share is the highest in the lower income quintiles, varying between 81–89 % of total household health care expenditure, while the share is the lowest (70–79 %) in the higher income quintiles. Expenditures on pharmaceuticals and medical devices increase from 2.9 % in 2005 and 2006 to 3.7 % of net household income in 2008.

Formal payments for health care services account for 0.5-0.6 % of household income. Its share in total household expenditure on health care is 13 % in 2005 and 2006, which increases to 15 % in 2007 with the introduction of co-payments for health care services, and decreases to 11 % in 2008 with the abolishment of these fees. Households in the lowest income quintile pay a 3.2-3.6 times higher share of their income on these items. The share of the expenditures on formal payments is the highest in the highest income quintile (17–22 %), while it is lowest in the lowest income quintile (7–13 %). Households from the highest income quintile pay only a 1.2-1.9 times higher share of their income on these items than households from the lowest quintile.

Informal payments account for 0.2-0.3 % of the household income. These payments represent 9 % of the total expenditure in 2005 and 2007, and decrease to 4 % and 5 % in 2007 and 2008 respectively. The share of these payments in total expenditure is rather equal among income quintiles (varying between 8–10 % before the reform period, and 4–6 % after the reform.)

**Figure 1:**
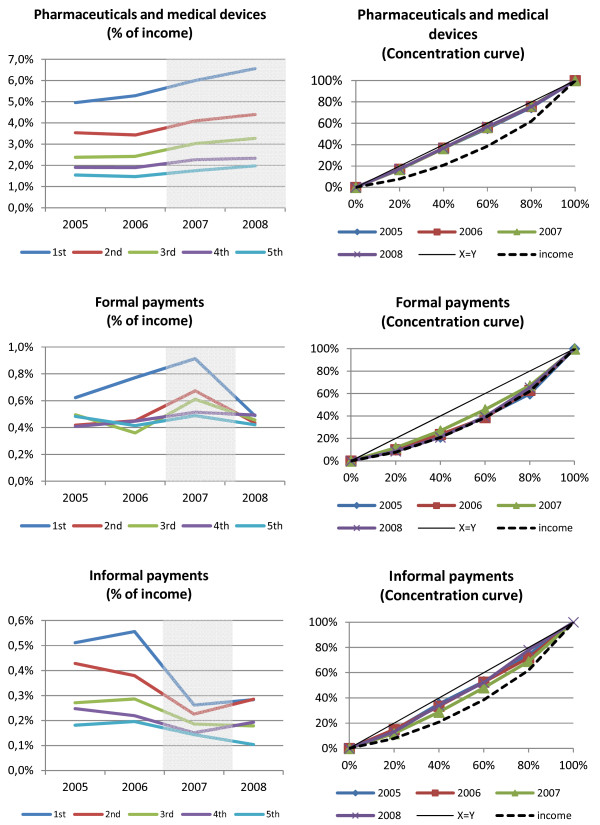
The trend and concentration of the three types of household expenditure on health care.

In Figure 1, the charts on the left side illustrate the trend of the share of different types of health care expenditure as a percentage of income. On the charts on the right side, the concentration curves of the different types of expenditures graphically illustrate how the expenditures are distributed across the households ranked by income for the examined years (2005–2008). The concentration curve plots the cumulative percentage of different types of expenditures (and also income) on the vertical axis against the cumulative percentage of households ranked by their household income on the horizontal axis, beginning with the poorest and ending with the richest. ^d^

Table 3 presents the value of the Kakwani index for the different types of health care expenditure. The negative values of the Kakwani index indicate that the distribution is regressive for the total household expenditure on health care, approximately −0.22 in all four years examined. This indicates that, lower-income households spend a higher share of their income on health care than better-off households.

**Table 3:** Kakwani indexes by type of expenditures

**Kakwani index by type of expenditures**	**2005**	**2006**	**2007**	**2008**
Pharmaceuticals and medical devices	−0.235*** (−32.03)	−0.238*** (−32.39)	−0.240*** (−31.11)	−0.233*** (−30.99)
Formal payments for health care services	−0.004 (−0.19)	−0.024 (−1.21)	−0.096*** (−5.13)	−0.009 (−0.36)
Informal payments for health care services	−0.203*** (−10.04)	−0.182*** (−8.64)	−0.121*** (−5.07)	−0.200*** (−5.48)
Total expenditure on health care	−0.220*** (−32.79)	−0.224*** (−33.00)	−0.220*** (−29.94)	−0.215*** (−29.44)

Note: *** p < 0.01; t-statistics are in parenthesis.

The reform period (year 2007) is indicated by bold letters.

The lowest value of the Kakwani index is found for pharmaceuticals and medical devices, varying between −0.24 in 2005, 2006 and 2007 and −0.23 in 2008, which indicates that this is the most regressive type of payment. Graphically, the curve of the expenditures on pharmaceuticals and medical devices is close to diagonal.

The Kakwani index is close to 0 in the case of formal payments, i.e. the coefficient is not significantly different from zero in 2005, 2006 and 2008, and significantly different from 0 in 2007 with the value of −0.10. This indicates proportionality of formal payments in 2005, 2006 and 2008. The graphs also show that formal payments for health care services follow the income (Lorenz) curve.

Expenditures on informal payments are regressive as well. The Kakwani index for informal payments varies between −0.18/-0.20 before and after the reforms. Its value increases to −0.12 in 2007.

From Figure 1, we can also visually observe the changes in the expenditures during the examined period. We observe no relevant changes in the progressivity of the payments during the examined period concerning expenditures on pharmaceuticals and medical devices. However, we do observe that in 2007, with the introduction of co-payments, the curve of the expenditures on formal payments diverges from the diagonal, which indicates that these payments become more regressive. However, the curve converge to the diagonal again in 2008 (which indicates proportionality of the expenditures), after the abolishment of co-payments. The curve of the expenditures on informal payments converges to the diagonal in 2007 compared to the other years examined, which indicates that informal payments became less regressive in 2007.

## Discussion of the results

We find that the burden of household expenditure on health care is not equally distributed among different income groups. Households from the lowest income quintile pay an about three times larger share of their income on health care compared to households in the highest income quintile.

Our results confirm the conclusion of previous studies, which found that out-of-pocket payments are a regressive means of raising health care revenues [2-5]. Examining some OECD countries, Wagstaff et al. (1999) found that out-of-pocket payments are most proportional to income in Germany and the Netherlands (Kakwani index higher than −0.1 %) and the highest in Switzerland (−0.36) and France (−0.34) (similar to the US (−0.38)) where the poor without insurance coverage rely mostly on out-of-pocket payments [3]. We find that the Kakwani index of total household expenditure on health is approximately −0.2 during the examined period in Hungary, which is comparable to the findings for the United Kingdom and Finland [3,28].

Concerning the neighboring countries and countries from the Central-European region, in Slovakia the Kakwani index of out-of pocket payments is −0.25 in 2004 and −0.23 in 2005 [29], which is comparable to our findings for Hungary. In Croatia, even higher inequalities can be found. Persons from the lowest income deciles pay an about six times larger share of their income on health care than the highest income deciles [5]. However, in the Central European region, the Czech Republic is an exception, where the share of out-of-pocket payments as a percentage of household income is much lower than found in our study (1.9 % in 2007, 2.2 % in 2008) and it is also distributed quite evenly across households [30].

Two previous studies reported Kakwani indexes of the out-of-pocket payments in Hungary [10]. Szende et al. (2002) find Kakwani indexes of −0.28 in 1999 and Csaba (2007) −0.27 in 2007 [31,32]. These values are slightly lower than those that we find in our study. The difference might be explained by differences in the methodology as previous studies calculated the index based on individual-level data, while we use expenditure data on household level. These studies however focus on data from one year and do not examine the trends in the equity of the payments.

We find that household expenditures on pharmaceuticals and medical devices are the most regressive types of expenditures (the lowest income quintile pays 3.6 times more as a share of income than the highest quintile in 2007). At the same time, these payments represent the major share of the total household expenditure on health care (75–85 %). The lack of adequate protection mechanisms in the pharmaceutical subsidy system might be the main reason for this finding. In Hungary, no ceiling for co-payments for pharmaceuticals and medical devices is applied, and the co-payments do not depend on income either. However, there is a rather controversial system for exemptions of vulnerable social groups from such co-payments. Some patients with disability or with an income below a certain level ^e^ are eligible for exemption from paying co-payments. During the examined period relevant changes took place in the exemption system. Since July 2006, for patients who are eligible for exemptions a certain budget is defined by their GP based on their medical needs. This budget should not exceed 12 000 HUF (~ 44 euros) per months. (Before, there was a list of pharmaceuticals and medical devices which were available free of charge for patients with a special certificate, without any limitation of the quantity or the amount). ^f^ However, according to the monitor of the National Auditory Office the number of patients eligible for exemption because of their income decreased by 56 % from 2005 to 2006, moreover the office found the system non-transparent, exemptions are not based on the real needs [33]. Our results suggest that protection of vulnerable social groups should be revised and extended to improve equity of these payments. Nevertheless it has been shown in previous studies that the lack of exemption mechanisms can produce inequalities in access to health care, which can lead to higher morbidity, emergency care admissions and mortality [34,35]. Here, we have to highlight that the health status of the Hungarian population is already lagging behind other European countries [10].

The high share of expenditures on pharmaceuticals and medical devices (which was observed in Hungary) is a common characteristic observed in most of the Central and Eastern European countries that joined the EU after 2004. Here, the expenditures on pharmaceuticals and medical devices account for more than 70 % of total health expenditure, while this share is below 50 % in the EU15 countries [36]. In Central and Eastern European countries, medicines are rarely included in the basic package, so patients are obliged to pay out-of-pocket. The relatively low labour cost in these countries could hold the prices of services low even in the private sector in contrast with the price of pharmaceuticals and medical devices, which are comparable with those in Western European countries.

Payments for health care services represent a minor share of total household expenditures on health care. According to our results around 20–40 % of these payments are informal payments for health care services. We find that expenditures on informal payments are regressive, which confirms previous results of Szende and Culyer (2006) on informal payments in Hungary [37]. This implies that the amount of informal payments initiated by patients or requested by medical staff is not related to the patients’ ability to pay. Thus, expenditures on informal payments impose a relatively higher burden on worse-off households, and might lead to inequalities to access.

However, expenditures on formal payments are found to be the most proportional to income. The explanation of this finding might be that before the introduction of co-payments in 2007 (as well as after the abolishment of the fees), most of the health care services covered by the social health insurance could be used free of charge and formal payments were mostly paid for private services. We assume that households with higher income use proportionally more private services. The finding that the expenditures on formal payments increase proportionally to income indicates that the private health care services are seen as a luxury goods which is in accordance with previous findings [38].

Based on previous results, we expect that that health care reforms, which increase the role of out-of-pocket payments in health care financing, lead to a relatively greater burden (of private payments) falling on the low income groups [5-7]. We find the same in the case of expenditures on formal payments. Compared to the observed proportionality in the previous years in 2007 when co-payments for public health care services were introduced, expenditures on formal payments became more regressive despite of the application of exemption categories (which were mainly based on the type of care not on income situation) and a stop-loss.

On the other hand, we find that despite of the increase of the co-payments for pharmaceuticals, the progressivity of expenditures on pharmaceuticals and medical devices did not change during the reform period. Thus, they remain as regressive as before the reforms.

Finally, we have interesting findings concerning household expenditure on informal payments. In CEE countries the introduction of co-payments is often motivated by their potential to eradicate or formalize informal payments [39-41]. This was also the case in Hungary where one of the main aims of the introduction of official user fees (besides the objective of curbing the unnecessary use of health care services) was to eradicate informal payments [42]. In this study, we observe a decrease in informal payments of households in parallel to the increase of formal payments. Also, this expenditure became less regressive during the reform period (i.e. the decrease was higher in the lower income households). This finding might suggest that worse-off households tried to compensate the increasing burden of formal co-payments with decrease in their expenditure on informal payments. However, we cannot really measure the causality of this association. Furthermore, the improvement observed in the equity of informal payments might also be the resultant of the larger drop in the utilization of services among worse-off households, which leads to less informal payments. Further research is needed to clarify the relationship between formal and informal expenditure.

## Discussion of the limitations

We have to acknowledge some limitations of our study. First, we have to highlight that the National Household Budget Survey does not contain data on the utilization of services. However, according to previous literature, the reform arrangements have resulted in major changes in the utilization of health care [43-45]. Due to the lack of utilization data in the dataset, we cannot quantify the effect of the increase in co-payments on the access to health care across households with different income. However, it is possible that in worse-off households the drop in utilization is higher as based on previous studies they are assumed to be the most sensitive to price changes [46,47]. In this case, we underestimate the increase of the burden in these households. To have a better insight in the effect of the reforms on equity, equity in access to the services should be also considered and should be the topic further research.

We have to highlight that for the calculations we use a data on household level. This might result in slight differences with previous results from Hungary [31,32] which are based on individual data.

Finally, we also have to consider that during the examined period, multiple, simultaneous reforms (in and outside of the health care sector as a result of the Convergence Program) took place in Hungary. It is difficult to differentiate between the effects of separate reform arrangements on the household expenditure.

## Conclusion and policy recommendations

In this paper, we have examined the progressivity of households’ out-of-pocket payments and the distribution of these payments across income quintiles between 2005–2008: before, during and after the period of health care reforms in Hungary (2006–2007). In 2007, comprehensive health care reforms took place in the country, with arrangements aimed at shifting costs to consumers (e.g. the increase of the co-payments for pharmaceuticals, the introduction of co-payments for health care services).

Expenditures on pharmaceuticals and medical devices, are the most regressive payments and represents the highest share of households’ health care expenditure (more than 75 %). However the progressivity of these payments has not changed during the reform period. Future health care reforms should consider the improvement of the equity of these payments by the improvement of protection mechanisms i.e. the introduction of a maximum limit for co-payments for pharmaceuticals and medical devices or the implementations of exemption mechanisms of vulnerable social groups.

We find that in the case of formal payments, changes such as the introduction of co-payments for public health care services, led to a relatively greater burden falling on low income groups despite of the application of exemption categories and a stop-loss. These effects on access to health care services should be carefully considered before the increase/introduction of co-payments and special attention should be paid to the protection of vulnerable households from an increased burden on their household budgets. It is necessary to assure access to health care services for those who are not able to pay for the services. Our results also suggest that households, especially in the lower income deciles tried to compensate the increasing burden of health care expenditure by decreasing expenditures on informal payments. However, further research is needed to study the causality of these relations.

The results of our study might serve as a useful example for other European countries, where the expansion of patient cost-sharing is considered. Especially in the Central and Eastern European countries that joined the EU in and after 2004, since in these countries, the structure, financing and operation of the health care system are similar to those in Hungary. Furthermore, in the last decades, these countries all considered or have been considering an increase of out-of-pocket payments to cope with the continuous deficit of the health insurance funds, and financial difficulties of health care providers.

More attention should be paid to the protection of vulnerable social groups (in particular the poor) when implementing patient charges (especially for pharmaceuticals but also for services). Also, it is important to eliminate the practice of informal payments in order to improve equity in health care financing and to avoid inequalities in access to health care.

## Endnotes

^a^ See more details on the survey here: http://portal.ksh.hu/pls/ksh/ksh_web.meta.objektum?p_lang=EN&p_menu_id=110&p_ot_id=100&p_obj_id=ZHC&p_session_id=48488026; ^b^ See more details on the classification here, http://stats.oecd.org/glossary/detail.asp?ID=352; ^c^ See more details here: http://portal.ksh.hu/pls/ksh/ksh_web.meta.objektum?p_lang=EN&p_menu_id=410&p_almenu_id=101&p_ot_id=100&p_level=1&p_session_id=15276620&p_obj_id=ZHC; ^d^ See more on concentration curves in Quantitative Techniques for Health Equity Analysis—Technical Note #6 http://siteresources.worldbank.org/INTPAH/Resources/Publications/Quantitative-Techniques/health_eq_tn06.pdf; ^e^ Disabled and those persons are enabled for the certificate whose medical expenses exceed 10 % of the minimum pension and the family income per person do not exceed the minimum pension (in 2010 around 100 euros) or 150 % in case of the person is living alone. ^f^ See more details here: http://www.oep.hu/pls/portal/docs/PAGE/LAKOSSAG/OEPHULAK_EBELLAT/ACH%C3%8DVUM%202010/KOZGYOGYELLATAS.PDF.

## Competing interest

The authors declare they have no competing interests.

## Authors’ contribution

PB have made substantial contributions to conception and design, carried out the analysis and drafted the manuscript. MP had contributions to the design, been involved in the analysis and helped to draft the manuscript. LG was involved in the formulation of the research question, in the introduction and background section as well as in the discussion of the results in Hungarian policy context. WG made substantial contributions to conception and design and critically revised the manuscript. All authors read and approved the final manuscript.

## Authors’ information

BP is a PhD researcher at Maastricht University, Teaching Assistant at Corvinus University of Budapest, and junior researcher at Center for Public Studies Affaires Foundation. MP is as assistant professor at Maastricht University, the Department of Health Services Research; CAPHRI; Maastricht University Medical Center; Faculty of Health, Medicine and Life Sciences. GL is professor at Corvinus University of Budapest, where he is the head of the Health Economics and Health Technology Assessment Research Centre. GL is also a senior researcher at Center for Public Studies Affaires Foundation. Wim Groot is a professor at Maastricht University, the Department of Health Services Research; CAPHRI; Maastricht University Medical Center; Faculty of Health, Medicine and Life Sciences.
